# Machine Learning–Driven Discovery of Sustainable Ionic Liquids for CO_2_ Capture from Large Chemical Spaces

**DOI:** 10.1021/acs.jpcb.6c02062

**Published:** 2026-07-13

**Authors:** Yushan Chen, Yongsheng Chen

**Affiliations:** Department of Civil and Environmental Engineering, 1372 Georgia Institute of Technology, 790 Atlantic Drive NW, Atlanta, Georgia 30332, United States

## Abstract

Ionic liquids (ILs) represent one of the most promising and versatile solvent families for CO_2_ capture due to their structural tunability and low volatility. However, the sheer scale and complexity of the IL chemical design space make the discovery of high-performance candidates extraordinarily challenging. In this work, we introduce a SHAP-guided Domain Filter (SGDF) framework that leverages machine learning (ML) to address this challenge, enabling reliable large-scale screening across millions of potential IL structures. Starting from over 32 million cation–anion combinations, sequential SGDF gates based on melting point (*T*_m_), toxicity, viscosity, and CO_2_ solubility reduce the search space by more than 2 orders of magnitude, ultimately yielding 69,009 promising candidates. Experimental validation is performed on 12 ILs, including two model-selected candidates that exhibit the desired properties. Notably, the two model-selected ILs exhibit the highest CO_2_ absorption capacities, surpassing all other samples. Across the 12 ILs evaluated, the framework achieves high predictive accuracy (RMSE = 0.046, MAE = 0.030, *R* ^2^ = 0.781), showing excellent agreement between predicted and experimental results. These findings demonstrate that combining predictive modeling with experimental validation offers both mechanistic insight and a scalable, data-driven pathway for designing next-generation sustainable ILs for CO_2_ capture.

## Graphical abstract

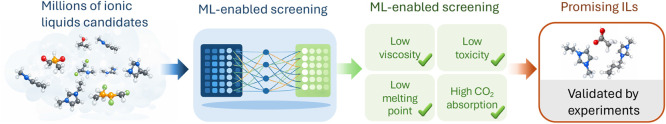

## Introduction

The urgent need to mitigate climate change has led to intensive efforts to develop efficient and scalable carbon capture technologies.^1^ Among these, ionic liquids (ILs) have attracted significant attention as next-generation absorbents due to their unique physicochemical properties,^2^ such as negligible vapor pressure,^3^ high thermal stability,^4^ and tunable CO_2_ solubility.^3^ However, the very structural flexibility that gives ILs their desirable properties also leads to an immense and complex chemical design space.^5^ This vast chemical space arises from the combinatorial possibilities of various cation–anion pairs and functional groups, which creates great potential for CO_2_ absorption.^6^ At the same time, the diversity of potential possibilities represents a core challenge, making it nearly impossible to explore vast candidates through conventional experimental methods.^7^ The challenge, therefore, is to shift from inefficient experimental screening to computational modeling for the identification of ILs that achieve high CO_2_ uptake while maintaining low viscosity, low melting point (*T*_m_), and low toxicity.

Traditional approaches to modeling IL–CO_2_ interactions have primarily relied on thermodynamic,^8^ molecular simulation,^9^ and computational chemistry frameworks.^10^ Empirical correlations such as the extended Henry’s law, activity coefficient-based models, and group contribution methods^11^ have been widely adopted to link IL structure to CO_2_ solubility. While effective for interpolating and extrapolating within structurally similar IL families,^12^ they suffer from fundamental limitations. Despite their theoretical rigor, traditional computational frameworks are hindered by prohibitive computational costs and poor scalability. These methods require intensive resources and extensive sampling times to achieve statistical convergence, particularly for viscous ionic liquid systems. Consequently, their application is often restricted to small-scale molecular systems or limited sets of candidates, making them computationally impractical for the high-throughput screening required to navigate the vast chemical space of potential ILs. Density Functional Theory (DFT) is frequently employed to calculate interaction energies, optimized geometries, and charge distributions between the IL ion pair and the CO_2_ molecule.^13^ Conductor-like Screening Model for Real Solvents (COSMO-RS) relies on quantum chemical descriptors to estimate thermodynamic properties by treating the solvent as a continuum and considering surface charge density.^14^ Although these methods capture the subtle electronic and solvation effects dictating interactions, they are computationally expensive, often restricting studies to isolated ion pairs or small clusters, and are intractable for bulk-phase or long-term dynamic simulations. Molecular Dynamics (MD) simulations offer atomistic resolution of the liquid state, allowing for the investigation of CO_2_ transport and absorption kinetics as well as microscopic structure.^15^ MD is crucial for calculating transport properties like diffusivity and viscosity, which are vital for process design.^16^ However, the accuracy of MD results is highly dependent on the quality and transferability of the underlying force field parameters.^17^ Furthermore, accurately sampling the complex phase space and reaching the equilibrium solubility state often necessitates microsecond-scale simulations for numerous state points,^15^ contributing to a prohibitively high computational cost that effectively limits their application to a small fraction of the vast IL design space.

To address these limitations, machine learning (ML) has emerged as a transformative paradigm for IL discovery. By leveraging existing experimental data sets, ML models can capture complex, nonlinear relationships between IL structure and relative properties, offering both rapid prediction and chemical insight.^18^ In recent years, ML has been used to estimate CO_2_ solubility, viscosity, and toxicity with encouraging accuracy.^19^ Nevertheless, challenges remain: many ML models function as “black boxes” with limited interpretability;^20^ in addition, extrapolation to unexplored regions of chemical space often leads to unreliable predictions.

In this study, we present an interpretable and experimentally validated ML framework for the discovery of environmentally benign ILs for CO_2_ capture. Our methodology designs a novel SHAP-guided Domain Filter (SGDF). This SGDF framework utilizes dual trust-zone criteria (Dice and Tanimoto similarity) to ensure screened candidates reside within the model’s chemical validity domain and finish preselection, bridging physical interpretability with data-driven confidence. Starting from an enumeration of 32 million cation–anion combinations, our sequential screening across melting point, toxicity, viscosity, and CO_2_ capacity effectively reduced the search space by more than 2 orders of magnitude. To verify the predictive reliability, we experimentally tested 12 ILs, ten commercially available comparators, and two model-selected candidates. Experiments validate the model’s accuracy (RMSE = 0.046, MAE = 0.030, *R* ^2^ = 0.781) and demonstrate that interpretable ML coupled with experimental verification provides a credible path toward IL discovery. This work contributes a methodological advance by establishing an interpretable, domain-aware ML pipeline capable of quantifying structure–property relationships at the fingerprint level. Practically, it demonstrates the power of this approach in accelerating sustainable solvent design. By combining explainable learning, domain-aware filtering, and targeted experimental validation, the proposed SGDF framework provides a scalable and generalizable route toward advancing carbon capture technologies and mitigating greenhouse gas emissions.

## Methods

The overall methodological framework of this study is summarized in Figure 1. The workflow integrates data collection, machine learning model development, model interpretation and trust-zone construction, large-scale screening of IL candidates, and experimental validation. In the following sections, we describe each component of the framework in detail, including the data sets, modeling strategies, interpretability analysis, screening criteria, and experimental procedures.

**Figure 1:**
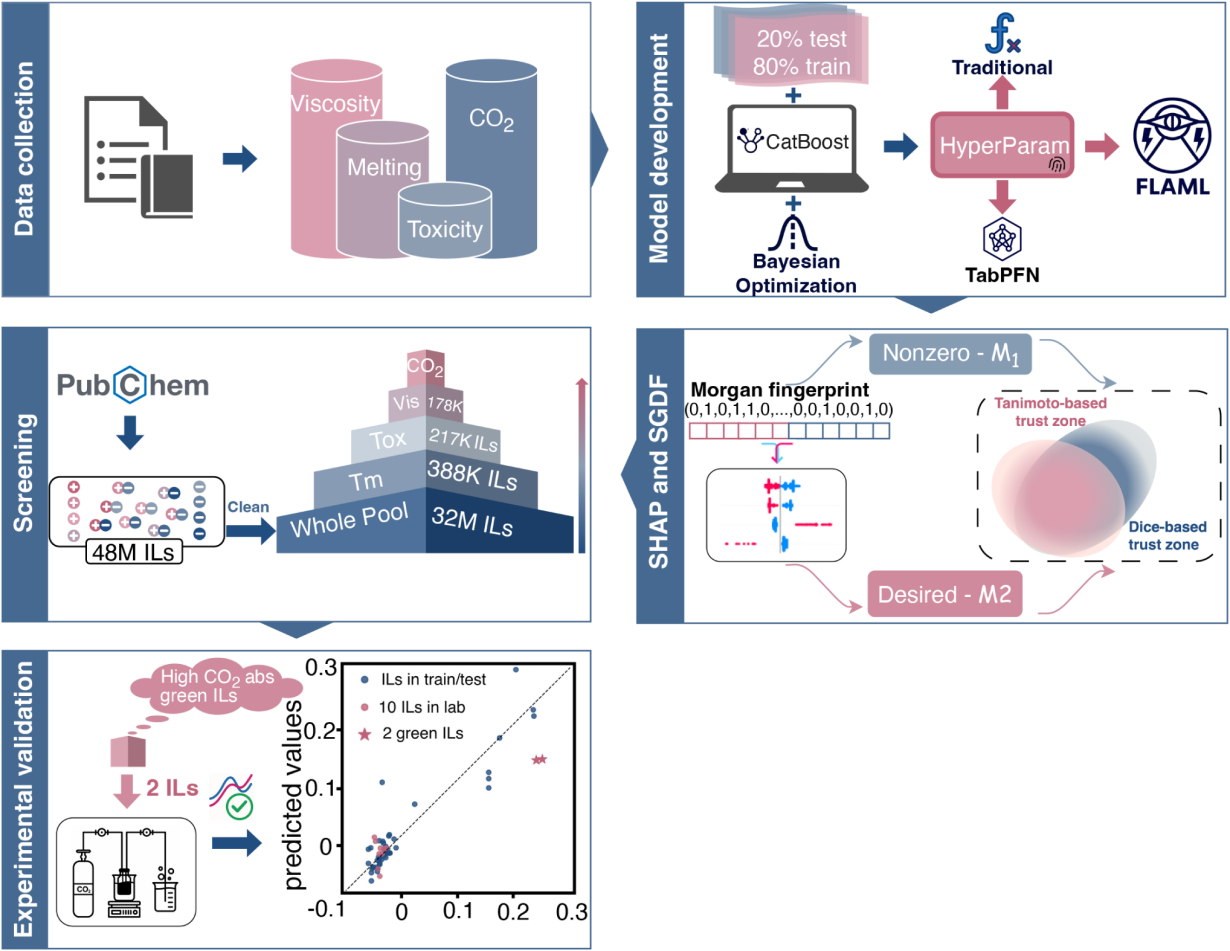
Pipeline illustration of the SGDF framework, encompassing data collection, model development, SHAP-guided interpretation, large-scale screening, and experimental validation. Starting from approximately 32 million ionic liquids, sequential SGDF filters on melting point, toxicity, viscosity, and CO_2_ capacity reduce the candidate pool to 69,009 promising structures. Experimental validation on 12 ionic liquids, including two model-selected candidates, confirms the framework’s predictive reliability.

### Data

#### Experimental Data Sets

The experimentally measured properties of the ILs were compiled from the NIST Ionic Liquids Database (ILThermo) for four targets relevant to carbon capture and deployment: viscosity, toxicity, *T*_m_, and CO_2_ solubility.^21^ The number of records for each property is summarized in Table 1, and all values were converted to consistent units. Duplicate values were collapsed via median aggregation; outliers were flagged using robust thresholds and removed if inconsistent between sources. The curated data set was then split into training (80%) and testing (20%) subsets.

**Table 1:** Summary of Experimental Datasets Used in This Study

Property	Representation	Sample size	Conditions
Viscosity	log_10_η (cP)	14,102	*T*; *P*
Toxicity	log_10_EC_50_	451	—
Melting point	Kelvin	2276	—
CO_2_ absorption	Mole fraction $\left(x_{CO_{2}}\right)$	14,711	*T*; *P*

#### ILs’ Structural Space

We constructed the ILs chemical space by enumerating random pairings between all human-curated cations and anions collected from PubChem.^22^ After deduplication and basic sanity checks (charge balance, valence/SMILES validity, and removal of fragments and salts), the resulting space comprises more than 32 million unique cation–anion combinations available for the following screening.

### Model Development

#### Feature Representation

IL structures were encoded as Morgan Fingerprints (MF), which were generated from canonical SMILES using RDKit.^23^ The fingerprint hyperparameters, primarily the radius and bit length, were optimized through a Bayesian hyperparameter search. A discrete bounded search space with 5-fold cross-validation (CV) was employed,^24^ where a CatBoost regressor was trained for each candidate setting. The best configuration was selected and fixed for all this properties’ subsequent modeling.

#### Model Setup

With the MF parameters fixed, we benchmark three pipelines under identical preprocessing; all scalers and encoders are fit on the training data only: (i) **Traditional**—a panel of standard regressors (e.g., CatBoost, XGBoost, Random Forest, etc.) under the same preprocessing, where CatBoost adopts the hyperparameters obtained from the Bayesian search described above and the remaining regressors are lightly adjusted; (ii) **AutoML (FLAML)**—a cost-aware joint search over estimator and hyperparameters on the training split, with a 1200 s time budget and a candidate pool including XGBoost, CatBoost, LightGBM, etc., optimizing mean squared error (MSE);^25^ (iii) **TabPFN**—applied to regression by discretizing the target into ordered bins and aggregating the predicted class probabilities into a point estimate.^26^ Model selection is performed by choosing the model with the highest *R* ^2^ on the test set.

### Model Interpretation and SGDF

After selecting the optimal pipeline and tuning the MF parameters, the best-performing model is interpreted using Shapley additive explanations (SHAP)^27^ to quantify the contribution of each MF bit to the predicted target. Using SHAP values, we ranked MF bits by their global importance for each target and separated directionality. For screening, we form a *property-specific active mask* $M_{n}$ that collects the bits aligned with the different directions of change. For properties whose models include temperature and pressure as input features (viscosity and CO_2_ absorption), predictions during screening were evaluated under fixed ambient conditions of *T* = 298.15 K and *P* = 101.325 kPa, consistent with the conditions of the experimental validation reported in this work.

#### Gate 1: SHAP-Activation Mask

From the training data, we construct a *property-specific active mask* $M_{1}$ consisting of MF bits with nonzero SHAP magnitude for the desired properties. For any candidate IL with MF bitset *A* and training-data-based IL *B* restricted to $M_{1}$ , we compute the Dice coefficient

$$\mathrm{Dice}\left(A_{M_{1}},B_{M_{1}}\right)=\frac{2\left|A_{M_{1}}\cap B_{M_{1}}\right|}{\left|A_{M_{1}}\right|+\left|B_{M_{1}}\right|}$$

We estimate a trust-region threshold θ_Dice_ as the 25th percentile of Dice similarity observed on the training split when each training IL exhibits property values consistent with the desired direction. Candidates in the enumerated IL structural space are retained if $\mathrm{Dice}\left(A_{M_{1}},B_{M_{1}}\right)\geq \theta_{\mathrm{Dice}}$ , thereby ensuring activation-level resemblance to chemistry seen in screening.

#### Gate 2: Direction-Sensitive Motif Filter

To further enforce the desired direction of effect, we construct a second mask $M_{2}$ comprising MF bits whose SHAP signs are consistent and stable for a given property: for *T*_m_ and viscosity we retain bits with *negative* contributions, whereas for toxicity (as log_10_EC_50_) and CO_2_ solubility we retain bits with *positive* contributions. For candidates that pass Gate 1 with bitset A, we compute the Tanimoto similarity to training-data-based IL B,

$$\mathrm{Tan}\left(A_{M_{2}},B_{M_{2}}\right)=\frac{\left|A_{M_{2}}\cap B_{M_{2}}\right|}{\left|A_{M_{2}}\cup B_{M_{2}}\right|}$$

We retained candidates whose minimum Tanimoto similarities, measured within the training split among structures that satisfied Gate 1, , exceeded the threshold. This enforces structural consistency aligned with the direction-sensitive motif pattern and achieves preselection before property prediction.

The two gates define an applicability domain tailored to properties: gate 1 guarantees sufficient activation overlap in the SHAP-relevant subspace, while gate 2 enforces global structural proximity consistent with the observed direction of effects. SGDF is applied sequentially to *T*_m_, toxicity, viscosity, and CO_2_ capacity, with the final set defined as the intersection of candidates meeting the desired directions.

### Experimental Validation

CO_2_ absorption experiments were conducted at atmospheric pressure and 25 °C using a bench-scale bubbling setup. Twelve ionic liquids were investigated; their chemical names, sources, and purities are provided in Supplementary Table S1. Carbon dioxide gas was purchased from Airgas.

CO_2_ was delivered from a compressed gas cylinder through a two-gauge pressure regulator and metered by a rotameter at a flow rate of approximately ∼100 mL min^–1^. In each experimental run, 10 mL of IL was placed in a sealed glass vessel and sparged with CO_2_ while being maintained isothermal in a thermostated water bath at 25 °C. Mass uptake was continuously tracked using a microbalance, and equilibrium was considered achieved when the mass increase between consecutive 10-min interval readings was less than 0.001 g. The final CO_2_ absorption capacity was recorded as the equilibrium mass increase. Each measurement was performed in triplicate; standard deviations of replicate measurements ranged from 0.0005 to 0.006 g, indicating satisfactory reproducibility. As ionic liquids are hygroscopic, traces of moisture or impurities may affect early uptake kinetics; however, prior work has shown that such impurities have negligible influence on equilibrium absorption capacity,^28^ and thus the reported end point values are considered reliable.

## Results and Discussion

### Integrated Model Evaluation

#### Model Performance

We evaluated three modeling setups for each of the target properties. First, a traditional suite of standard regressors with light hand-tuning under identical preprocessing. Second, an AutoML pipeline that performs cost-aware selection of estimators and hyperparameters while optimizing the MSE. Third, TabPFN, which treats regression as an ordered classification task and converts the predicted class distribution into a single numeric estimate.

As summarized in Figure 2a, FLAML attains the best test performance overall across the four targets: CO_2_ capacity, viscosity, *T*_m_, and toxicity, yielding the highest *R* ^2^, traditional regressors perform moderately, and TabPFN exhibits the weakest overall performance. This is likely because TabPFN is designed for smaller data sets; indeed, it performs relatively well for the smaller *T*_m_ and toxicity data sets, but its accuracy decreases for the larger viscosity data sets.^29^ Notably, for viscosity, the MF configuration with radius and length of (2, 4715) results in a substantially larger feature space compared with the other three data sets. Compared with the manually tuned traditional suite of regressors, the AutoML framework provides automated iterative hyperparameter optimization, which consistently results in superior performance across all four properties. Overall, FLAML delivers the most robust and stable results, and the FLAML-selected model is therefore adopted for downstream interpretation and screening.

**Figure 2:**
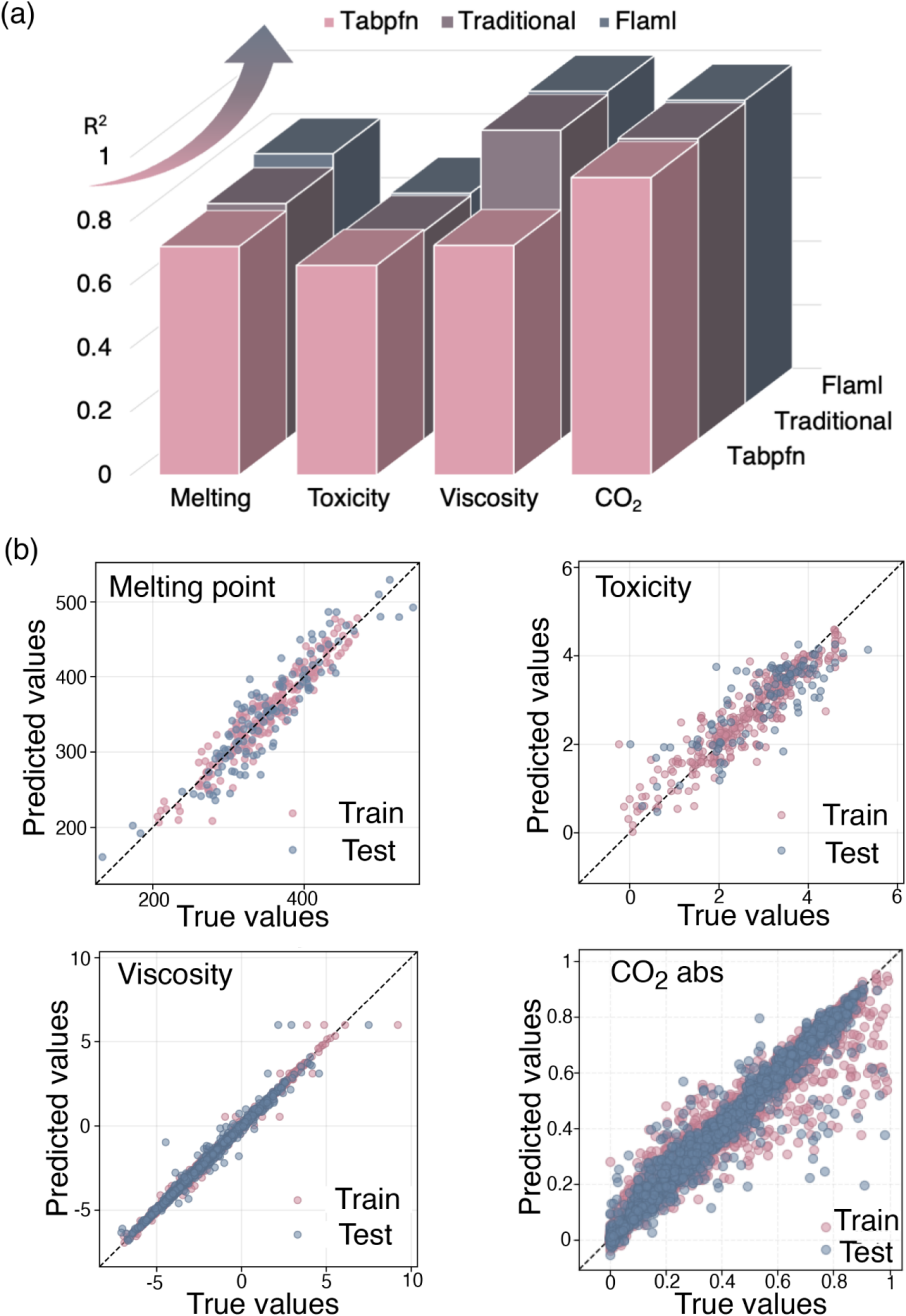
Model performance across four target properties. (a) *R* ^2^ comparison of traditional, TabPFN, and AutoML (FLAML) models; FLAML consistently achieves the highest *R* ^2^ across all properties. (b) Predicted vs true values for the four FLAML models, with both training and test points clustering tightly around the one-to-one line, confirming strong generalization.

All models achieve strong *R* ^2^ and MSE performance (Table 2); however, the MSE for the *T*_m_ model appears relatively large because the *T*_m_ data set spans a much broader numerical range (191–582 K) than the other targets.^30^ To better assess model accuracy under this broad scale, we additionally computed the Mean Absolute Percentage Error (MAPE), which is 1.2% for the training set and 4.1% for the test set, indicating that the model performs well despite the large absolute target scale. To further verify that the model captures meaningful variance rather than reverting to a trivial central tendency, we benchmarked it against mean, median, and random baseline predictors: the FLAML model reduces test MSE by 78.9% relative to the mean baseline and lowers test MAPE from 14.3% (mean baseline) to 4.1%. The full baseline comparison is provided in Supplementary Table S2. This conclusion is further supported by the visualization in Figure 2b, where both the training and test points cluster closely around the one-to-one line.

**Table 2:** Models’ Performance

Model	Train *R* ^2^	Test *R* ^2^	Train MSE	Test MSE
Vis model	0.993	0.983	0.020	0.044
Tox model	0.850	0.663	0.175	0.374
*T*_m_ model	0.975	0.786	91.833	820.720
CO_2_ abs model	0.979	0.951	0.001	0.026

#### Model Interpretation

To analyze how structural and thermophysical factors influence the four target properties, we employed a SHAP analysis to interpret the models. The top-ranked MF features were extracted by mean absolute SHAP value, and the corresponding substructures were visualized (Figure S2). The resulting attributions highlight chemically interpretable motifs and physical dependencies that align with well-established experimental knowledge (Figure 3), which also proves the reliability of our model.

**Figure 3:**
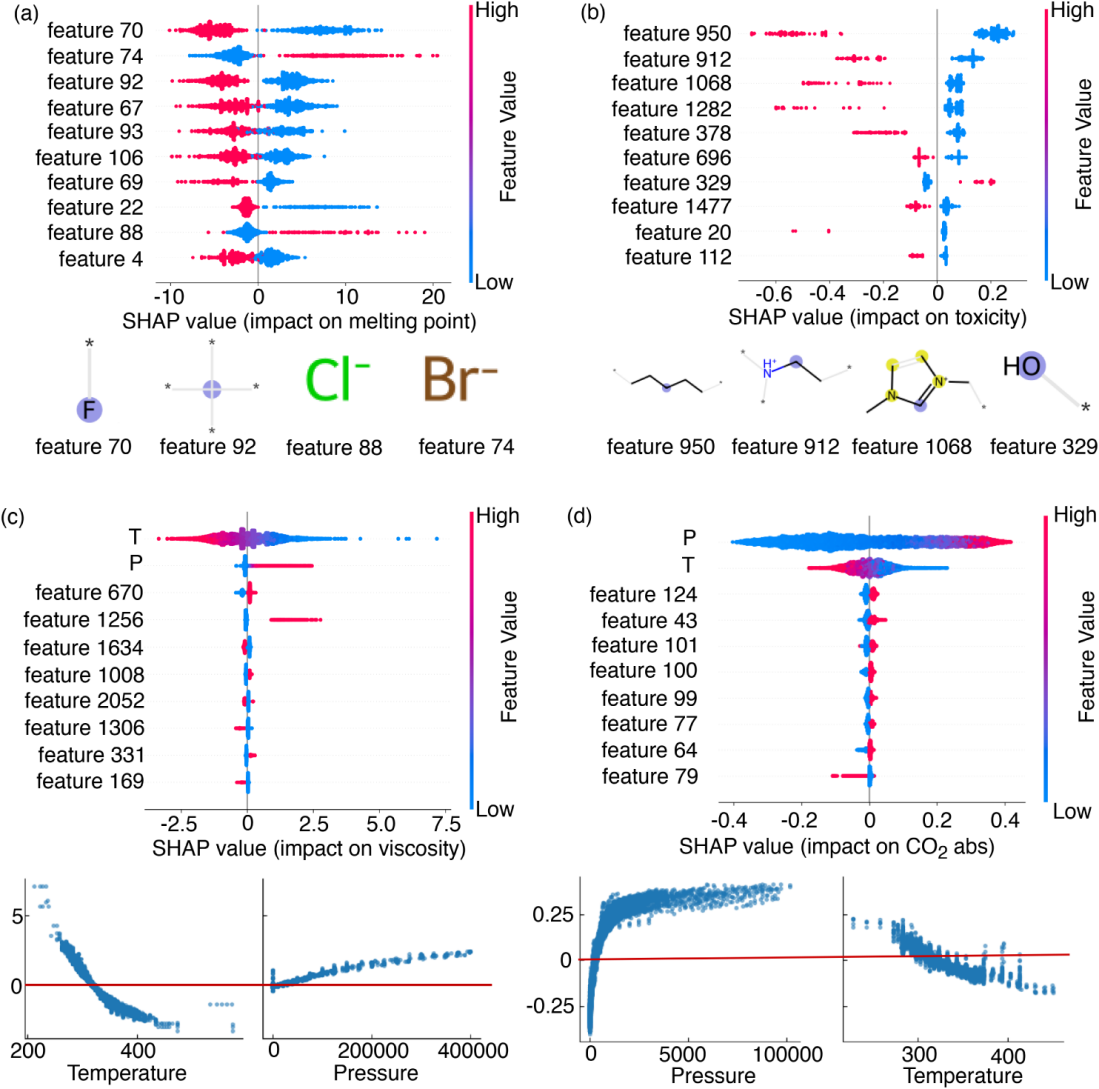
SHAP analysis results for the four target-property models. (a) *T*_m_ model: anion-related structural features dominate, with halide motifs contributing positively. (b) Toxicity model: aromatic cationic heterocycles (e.g., imidazolium, pyridinium) are the strongest positive contributors. (c) Viscosity model: *T* and *P* dominate over structural features; higher *T* decreases viscosity, while higher *P* increases it. (d) CO_2_ absorption model: *P* exerts the largest positive influence, while higher *T* reduces absorption.

For *T*_m_, the SHAP rankings in Figure 3a show that anion-related motifs dominate the feature space, with halide fingerprints exerting positive contributions by reinforcing ionic lattice cohesion and increasing crystal packing efficiency. These patterns align with experimental trends, where halide-containing anions promote stronger electrostatic interactions and restrict rotational freedom of ion pairs, leading to higher *T*_m_.^31,32^ Other weakly coordinating anions contribute negatively, reflecting their tendency to disrupt lattice order and lower melting points.^33^ These SHAP-derived patterns are consistent with findings from previous experimental studies, further supporting the reliability of the melting point model. Taken together, these attributions translate into a clear design rule: to achieve a low *T*_m_ suitable for room-temperature deployment, large and charge-delocalized anions are preferred over halide anions. In Figure 3b, cationic aromatic heterocycles such as imidazolium and pyridinium rings are identified as major positive contributors to toxicity, consistent with their known ability to penetrate lipid membranes and disrupt cellular structures.^34^ Conversely, hydroxylated or ether-containing fragments contribute negatively, indicating a detoxifying effect through increased polarity and reduced bioaccumulation potential.^35,36^ These attributions suggest that toxicity can be reduced by incorporating hydroxyl (−OH) into the cation side chain, while avoiding extended alkyl chains on imidazolium or pyridinium cations, as long hydrophobic chains are known to amplify membrane disruption.

For viscosity, Figure 3c shows that temperature (*T*) overwhelmingly dominates the SHAP contribution landscape, while pressure (*P*) has a secondary but consistent positive effect. The clear monotonic trend of decreasing viscosity with increasing temperature, where enhanced molecular mobility and weakened Coulombic interactions reduce viscous resistance.^37^ This agreement with experimental observations demonstrate that the model captures physically consistent dependencies without explicitly encoding them. Finally, for CO_2_ absorption capacity, Figure 3d reveals that pressure exerts the largest positive SHAP influence, directly consistent with Henry’s law behavior.^38,39^ In contrast, temperature shows strong negative contributions, reflecting decreased CO_2_ solubility at elevated temperatures due to weakened IL–CO_2_ interactions.^40^

Overall, the close correspondence between SHAP-derived attributions and experimentally established mechanisms across all four properties reinforces the model’s physical consistency and interpretability. This analysis not only validates the predictive framework but also highlights its potential as a discovery tool for guiding rational ionic liquid design through data-driven feature attribution.

### Large-Scale Screening Outcomes

#### IL Design Space

We initially constructed a chemical space of approximately 48 million unique cation–anion combinations through combinatorial enumeration. After removing duplicates and applying a series of fundamental validity checks, including charge balance, valence, and SMILES integrity, as well as the exclusion of fragments and salts, the space was refined to more than 32 million chemically valid ILs. These candidates were subsequently subjected to a hierarchical, sequential SGDF screening across *T*_m_, toxicity, viscosity, and CO_2_ capacity (Figure 4). A ML-guided screening framework is applied within a trust zone concept, shown in the dashed central box of Figure 4. Two complementary trust zones are defined: a Tanimoto-based trust zone and a Dice-based trust zone, ensuring that model predictions are restricted to regions of chemical space that are well supported by training data and thus physically reliable. Detailed domain filter criteria are provided in the Supporting Information.

**Figure 4:**
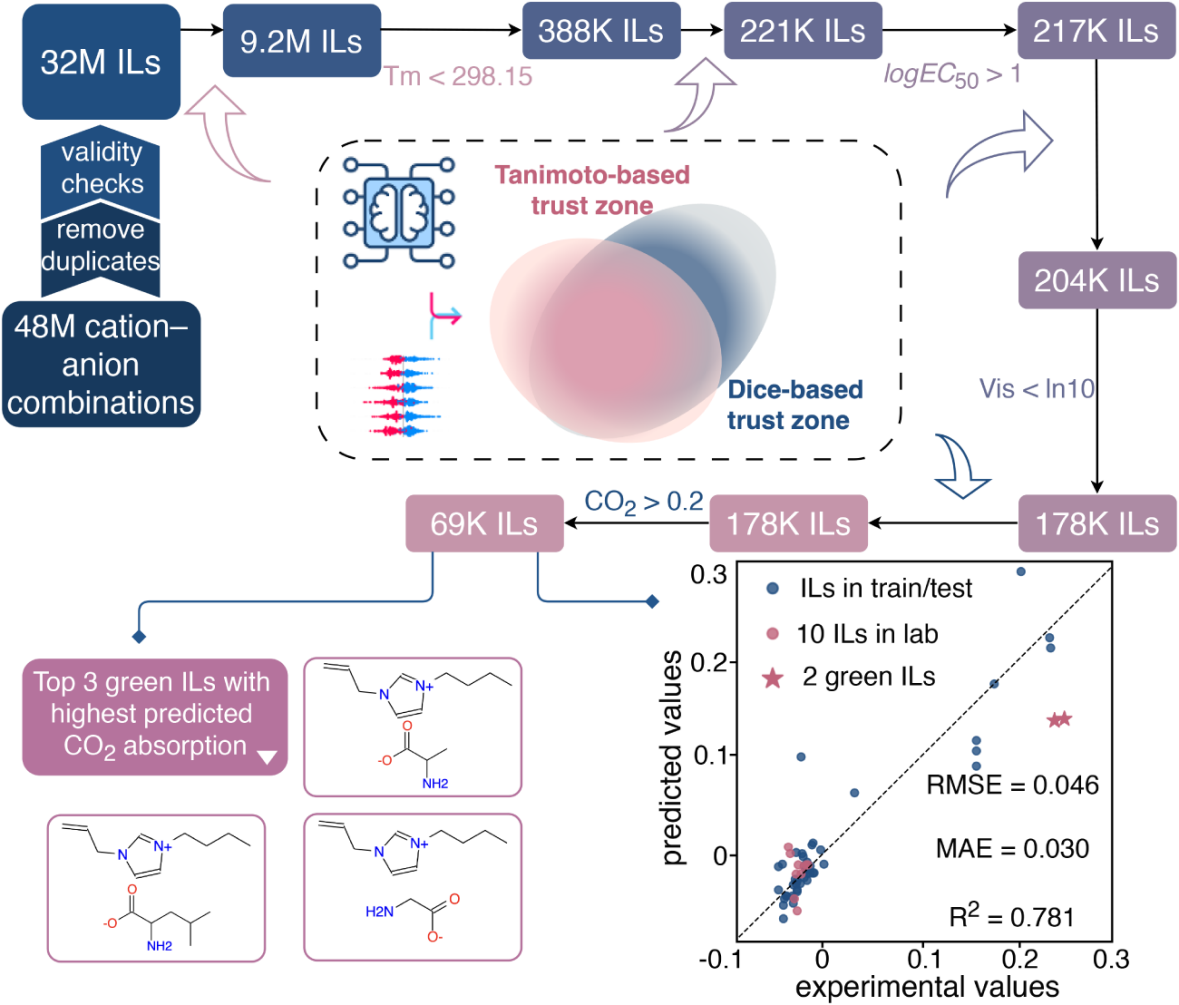
Large-scale screening process. Starting from 32 million ionic liquids, SGDF dual-gate filtering and sequential property constraints (*T*_m_ < 298.15 K, log *EC*_50_ > 1, viscosity < ln(10), CO_2_ > 0.2) reduce the pool to 69,009 candidates. The three top-ranked ILs by predicted CO_2_ absorption are highlighted, and experimental validation on 12 ILs confirms the framework’s reliability.

#### Sequential Multi-Property Screening

Following this, candidates were filtered by melting point (*T*_m_ < 298.15 K), reducing the candidate pool to approximately 388 K ILs. A subsequent toxicity screen based on the predicted log_10_EC_50_ > 1 further narrowed the pool to 217 K ILs. Additional viscosity constraints (viscosity < ln 10) reduced the candidate set to 178 K ILs. Finally, applying a CO_2_ absorption capacity threshold (CO_2_ > 0.2) yielded a compact subset of approximately 69009 ILs (Figure 4).

Overall, this hierarchical, multigate screening strategy reduced the initial chemical space by more than 2 orders of magnitude, ultimately identifying a compact set of promising sustainable IL candidates satisfying favorable requirements on melting point, toxicity, viscosity, and CO_2_ absorption performance. The complete SMILES representations and predicted physicochemical properties (*T*_m_, toxicity, viscosity, and CO_2_ capacity) for the top 100 selected candidates are provided in Github. To quantify the independent contribution of each SGDF gate, we conducted an ablation analysis across all four screening stages (Supplementary Table 3). Gate 1 (Dice similarity) provides the largest reductions in the early, broad-pool stages (22.7% and 37.5% for melting point and toxicity, respectively), while Gate 2 (SGDF score) delivers consistent additional refinement of 4.8–10.5% across all stages. This staged behavior is by design, as the framework front-loads aggressive filtering where chemical space is broadest.

#### Top-Ranked Candidates

Among the approximately 69,009 promising sustainable IL candidates retained after hierarchical screening, the molecular structures of the top three candidates with the highest predicted CO_2_ absorption capacity are shown in Figure 4. All three ILs are predicted to exhibit exceptionally high CO_2_ mole fractions (*x*CO_2_ > 0.64). Notably, none of these candidates have been previously synthesized or reported in the literature, highlighting the ability of the screening pipeline to uncover unexplored yet highly promising chemical motifs beyond conventional IL design paradigms.

#### Experimental Validation

Experimental measurements reveal strong quantitative agreement with model predictions across the full validation data set (Figure 4). On the basis of the 12 experimentally tested ILs, the predictive framework achieves an RMSE of 0.046, an MAE of 0.030, and an *R* ^2^ of 0.781, demonstrating robust accuracy despite the chemical diversity of the validation set.

To further characterize predictive performance, we conducted a residual analysis stratified by anion type. For ${\mathrm{BF}}_{4}^{-}$ -based ILs, the model performs with high fidelity (MAE = 0.012, bias ≈ 0), indicating that these structures fall well within the model’s applicability domain and are well represented in the training data. Sulfate-based ILs show a slightly larger error with a small positive bias (MAE = 0.020), suggesting modest extrapolation at the upper range of the training distribution. In contrast, acetate-based ILs exhibit systematic underprediction (MAE = 0.102), reflecting their structural divergence from the majority of training ILs and their position near the boundary of the model’s applicability domain. Importantly, this observation is consistent with the SGDF framework’s design intent: the dual-gate filtering mechanism is explicitly constructed to retain only candidates that reside within well-supported regions of chemical space. Accordingly, acetate-type ILs are largely excluded from the final 69,009-candidate pool by Gate 1 and Gate 2, and none appear among the top-ranked candidates. The elevated prediction error for acetate-based ILs therefore reinforces, rather than undermines the reliability of the screening outcome: the framework correctly identifies and deprioritizes structurally atypical candidates for which model confidence is lower.

Importantly, the validation is not limited to the nine ILs routinely used in our laboratories. To provide a broader and more representative assessment, we additionally compiled CO_2_ absorption data for ILs in the model training and test sets measured under ambient conditions. All available experimental and predicted data are jointly presented in Figure 4. Within this combined data set, the two ILs selected from the model-screened pool exhibit the strongest CO_2_ absorption performance among all experimentally tested samples, further supporting the reliability of the predictive framework and confirming the competitiveness of the newly identified IL candidates.

#### Implications and Methodological Advantages

The present framework also advances beyond recent ML-based IL screening efforts in scale, scope, and validation. Wang et al. built a SHAP-interpreted DNN model for IL-catalyzed CO_2_ cycloaddition yield prediction, identifying 14 promising candidates via ML-plus-DFT screening,^41^ while conventional screening studies demonstrated chemically informed active learning for multiobjective materials optimization.^42–44^ Relative to these studies, the present work operates over a substantially larger chemical space (32 million cation–anion combinations), simultaneously optimizes four deployment-relevant properties, and provides experimental rather than computational validation of out-of-distribution candidates—a combination that, to our knowledge, has not been demonstrated in prior IL screening work.

While the toxicity criterion (log_10_ EC_50_ > 1) effectively excludes acutely toxic structures, it does not capture broader environmental hazard dimensions such as persistence, bioaccumulation, or biodegradability; prior literature suggests that acetate-based ILs among the retained candidates tend to exhibit favorable biodegradability profiles.^35,36^ A comprehensive environmental assessment incorporating these factors remains an important direction for future work.

As a result, the proposed pipeline enables a more stringent and reliable down-selection from an immense chemical design space. These capabilities allow the model not only to accelerate the discovery of high-performance and sustainable ILs for CO_2_ capture, but also to provide insight into the structure–property relationships. Together, these results establish our screening pipeline as a scalable and generalizable paradigm for rational IL design and broader data-driven chemical discovery.

## Supplementary Material



## Data Availability

Code and complete screening results are available on GitHub at https://github.com/Yushan-Chen/ILs_opt_final.

## Notes

Table S1: ionic liquids used in this study, including chemical names, abbreviations, suppliers, and purities; Table S2: performance of the FLAML *T*_m_ model compared with trivial baselines on the test set; Table S3: ablation study of the SGDF dual-gate filtering framework across four sequential screening stages; Figure S1: similarity distributions for the four target-property models; Figure S2: top Morgan-fingerprint features by mean absolute SHAP value for each target-property model (PDF)
