# Supplementary material for: Machine Learning–Driven Discovery of Sustainable Ionic Liquids for CO2 Capture from Large Chemical Spaces
Source: J Phys Chem B. 2026 Jul 13;130(29):7424–32. doi: 10.1021/acs.jpcb.6c02062 (PMC13403316; doi:10.1021/acs.jpcb.6c02062)
Supplement: Supplementary file 1 [file jp6c02062_si_001.pdf]

Supporting Information for: Machine Learning–Driven Discovery of Promising Sustainable Ionic Liquids for CO_2 Capture from Large Chemical Spaces

Yushan Chen[†], Yongsheng Chen[†*]

[†]*Department of Civil and Environmental Engineering, Georgia Institute of Technology, 790 Atlantic Drive NW, Atlanta, GA 30332, United States*

E-mail: yongsheng.chen@ce.gatech.edu

Phone: +(404)894 3089. Fax: +(404)894 3089

Ionic Liquids Used in This Study

Twelve commercially available ionic liquids (ILs) were selected to span a broad range of cation and anion chemistries relevant to CO_2 capture applications (Table S1). All ILs were obtained from either Sigma-Aldrich or Tokyo Chemical Industry (TCI) at purities sufficient for spectroscopic and thermophysical characterization ($\geq$94%), and were used as received without further purification. This set provides representative coverage of the imidazolium-based IL chemical space and serves as the experimental basis for validating the ML model predictions.

Table S1: Ionic liquids used in this study, including chemical names, abbreviations, suppliers, and purities.

Chemical Name	Abbreviation	Supplier	Purity
1-Ethyl-3-methylimidazolium tetrafluoroborate	$[EMIM][BF_4]$	Sigma-Aldrich	≥98% (HPLC)
1-Butyl-3-methylimidazolium tetrafluoroborate	$[BMIM][BF_4]$	Sigma-Aldrich	≥98%
1-Hexyl-3-methylimidazolium tetrafluoroborate	$[HMIM][BF_4]$	Sigma-Aldrich	≥97.0% (HPLC)
1-Methyl-3-octylimidazolium tetrafluoroborate	$[OMIM][BF_4]$	Sigma-Aldrich	≥97.0% (HPLC)
1-Decyl-3-methylimidazolium tetrafluoroborate	$[DMIM][BF_4]$	Sigma-Aldrich	≥96.5% (HPLC)
1-Ethyl-3-methylimidazolium hydrogen sulfate	$[EMIM][HSO_4]$	Sigma-Aldrich	≥95.0%
1-Ethyl-3-methylimidazolium methyl sulfate	$[EMIM][MeSO_4]$	Sigma-Aldrich	≥98.0% (HPLC)
1-Ethyl-3-methylimidazolium ethyl sulfate	$[EMIM][EtSO_4]$	Sigma-Aldrich	≥95.0%
1-Ethyl-3-methylimidazolium trifluoromethanesulfonate	[EMIM][OTf]	Sigma-Aldrich	≥95.0% (^{1}H-NMR)
1-Ethyl-3-methylimidazolium bis(trifluoromethylsulfonyl)imide	$[EMIM][Tf_2N]$	Sigma-Aldrich	≥98.0% (HPLC)
1-Butyl-3-methylimidazolium acetate	[BMIM][OAc]	TCI	≥95.0% (HPLC)
1-Ethyl-3-methylimidazolium acetate	[EMIM][OAc]	TCI	≥94.0% (HPLC)

TCI = Tokyo Chemical Industry Co., Ltd.

Baseline Comparison for the T_m Model

To contextualize the absolute MSE of the T_m model, we benchmarked the FLAML model against three trivial baselines on the test set: a mean predictor (predicting the training-set mean for every sample), a median predictor, and a random predictor drawing uniformly from the training-set range (191–582 K, mean = 323.8 K, standard deviation = 60.3 K). As shown in Table S2, the FLAML model substantially outperforms all three baselines, reducing test MSE by 78.9% relative to the mean baseline (from 3883.3 to 820.7 K^2) and lowering the test MAPE from 14.3% to 4.1%. These results confirm that the large absolute MSE of the T_m model is an artifact of the broad numerical range of the target rather than an indicator of poor predictive performance.

Table S2: Performance of the FLAML T_m model compared with trivial baselines on the test set.

Model	Test R^2	Test RMSE (K)	Test MSE (K^2)	Test MAPE
Mean baseline	−0.011	62.316	3883.285	0.143
Median baseline	−0.070	64.102	4109.062	0.139
Random baseline	−4.384	143.785	20674.185	0.382
**FLAML (best)**	**0.786**	**28.648**	**820.712**	**0.041**

Similarity Distributions and SGDF Filtering Performance

To parameterize the SGDF, we derived two complementary masks. The $\mathcal{M}_1$ identifies Morgan-fingerprint (MF) bits with nonzero SHAP magnitudes for the target property. For each candidate IL, its Dice similarity with training ILs within $\mathcal{M}_1$ measures activation overlap, and the 25th percentile of these similarities defines the threshold θ_{Dice}, ensuring candidates remain in the SHAP-active subspace. The $\mathcal{M}_2$ retains bits with consistent SHAP signs—negative for T_m and viscosity, positive for toxicity and CO_2 capacity. Tanimoto similarity within $\mathcal{M}_2$ quantifies directional coherence, with θ_{Tan} as the secondary trust threshold. Candidates meeting both Dice $\geq \theta_{\text{Dice}}$ and Tan $\geq \theta_{\text{Tan}}$ define the dual-metric applicability domain, thereby also fulfilling the pre-selection requirement.

The resulting Dice and Tanimoto similarity distributions for all four properties are shown in Fig. S1. For all models, Dice similarities are higher than Tanimoto similarities, indicating that many ILs share SHAP-relevant structural features even if only a subset exhibit consistent directional effects. The distributions also reveal a strong dependence on MF length. Systems with shorter fingerprints, exemplified by T_m with a configuration of (4, 54), exhibit higher and more tightly clustered similarity values. As the fingerprint length increases through toxicity to viscosity with a configuration of (2, 4715), the similarity decreases in a systematic manner, with viscosity presenting the lowest values. This inverse relationship arises because longer fingerprints enlarge the union set $|A \cup B|$, reducing the intersection ratio $|A \cap B|/|A \cup B|$ and thus lowering both Dice and Tanimoto coefficients, consistent with previous analyses of fingerprint dimensionality and bit-density effects on molecular similarity.[1,2] In addition,

T_m and toxicity were used as the first-stage screening properties for the 32 million ILs, effectively removing candidates with rare or structurally atypical motifs. The subsequent filtering steps, guided by $\mathcal{M}_1$ and $\mathcal{M}_2$, further refined the candidate space within the SHAP-active chemical subspace. Overall, fingerprint length is the dominant factor governing the magnitude of molecular similarity, while $\mathcal{M}_1$ and $\mathcal{M}_2$ stabilize the screening process by excluding structurally inconsistent or unreliable candidates.

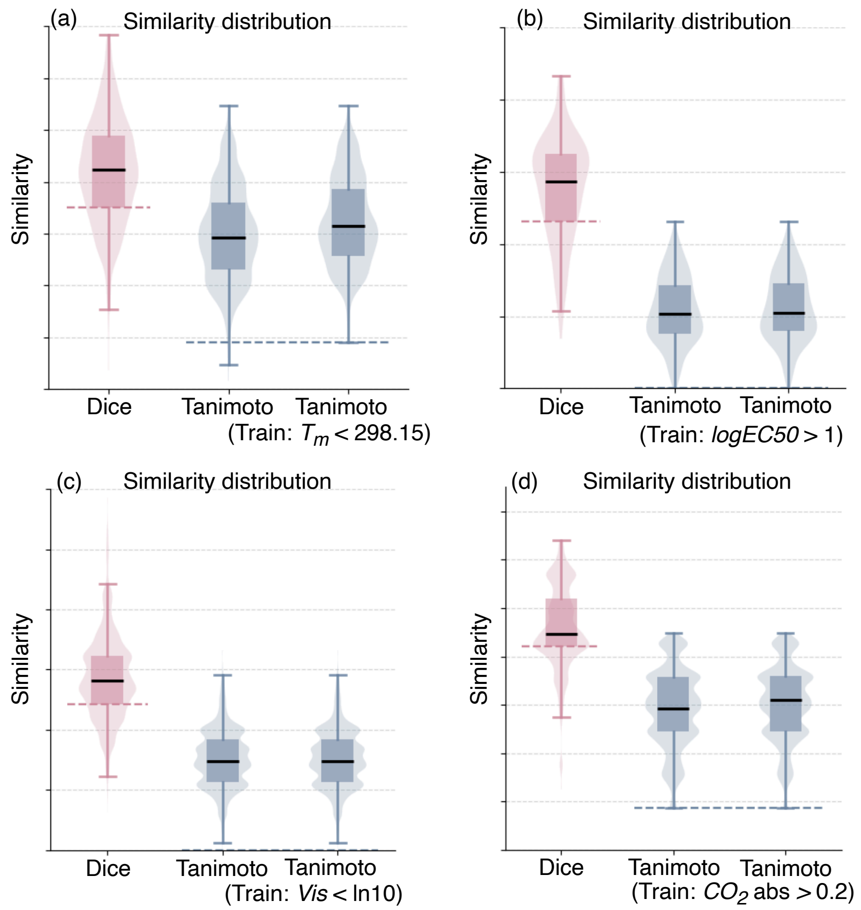

Figure S1: Similarity distributions for the four target-property models: (a) T_m, (b) toxicity, (c) viscosity, and (d) CO_2 capacity. The pink violin corresponds to Dice similarity within $\mathcal{M}_1$ (all training structures), and the blue violins represent Tanimoto similarity within $\mathcal{M}_2$ (desired-property subset only). Dice coefficients are consistently higher than Tanimoto coefficients across all models, reflecting both their mathematical definitions and the broader reference set used for Dice evaluation.

The quantitative impact of each gate on the candidate pool size is summarized in Ta-

ble S3. Gate 1 (Dice-based) alone eliminates a substantial fraction of structurally mismatched candidates at the melting-point stage, reducing the initial pool of 32.4 million ILs by approximately 22.7% to 25.1 million. The combined dual-gate filter (Gate 1 + Gate 2, SGDF) further removes an additional 2.6 million candidates, yielding 22.4 million ILs that are structurally coherent in both the SHAP-active and directionally consistent subspaces. For subsequent properties—toxicity, viscosity, and CO_2 capacity—the input pools are already substantially reduced by upstream screening, and the incremental attrition from Gate 2 is more modest, reflecting the fact that candidates surviving early filters are inherently more structurally aligned with the desired property subspaces. Collectively, these analyses confirm that the two SGDF gates define complementary aspects of the chemical trust zone: Gate 1 ensures activation-level overlap with the SHAP-relevant subspace, while Gate 2 captures directionally consistent motif alignment. They provide a property-adaptive, data-driven basis for reliable pre-selection before large-scale screening.

Table S3: Ablation study of the SGDF dual-gate filtering framework across four sequential screening stages. Input pool sizes reflect the number of candidate ILs entering each stage after all upstream filters have been applied.

**Property**	**Input Pool**	**After Gate 1 (Dice)**	**After Gate 1+2 (SGDF)**
Melting point	32,421,695	25,076,180	22,448,594
Toxicity	388,204	242,582	221,920
Viscosity	217,196	214,636	204,386
CO_2 capacity	178,527	178,519	178,426

SHAP Feature Analysis

To interpret the physicochemical drivers underlying each ML model, we extracted the top-ranked Morgan fingerprint features by mean absolute SHAP value and visualized the corresponding substructures (Fig. S2). For T_m, anion fragments dominate: fluorinated sulfonate and sulfonyl motifs (features 70, 92, 106) predict low melting points via charge delocalization and disrupted crystal packing, while halide anions (features 74, 88) carry opposing contributions. For toxicity, extended n-alkyl chain cation fragments (features 950, 912, 696,

et al.) dominate, consistent with the role of amphiphilicity in membrane permeability and cytotoxicity. For viscosity, key predictors span imidazolium and pyridinium cation fragments alongside fluorinated sulfonimide and dicyanamide anion motifs, reflecting the interplay of cation chain flexibility and anion coordination strength. For CO_2 solubility, multiple bis(trifluoromethylsulfonyl)imide anion variants (features 124, 101, 100, 99, 77) collectively dominate SHAP importance, consistent with the well-known CO_2 affinity of $[Tf_2N]^-$-based ILs.

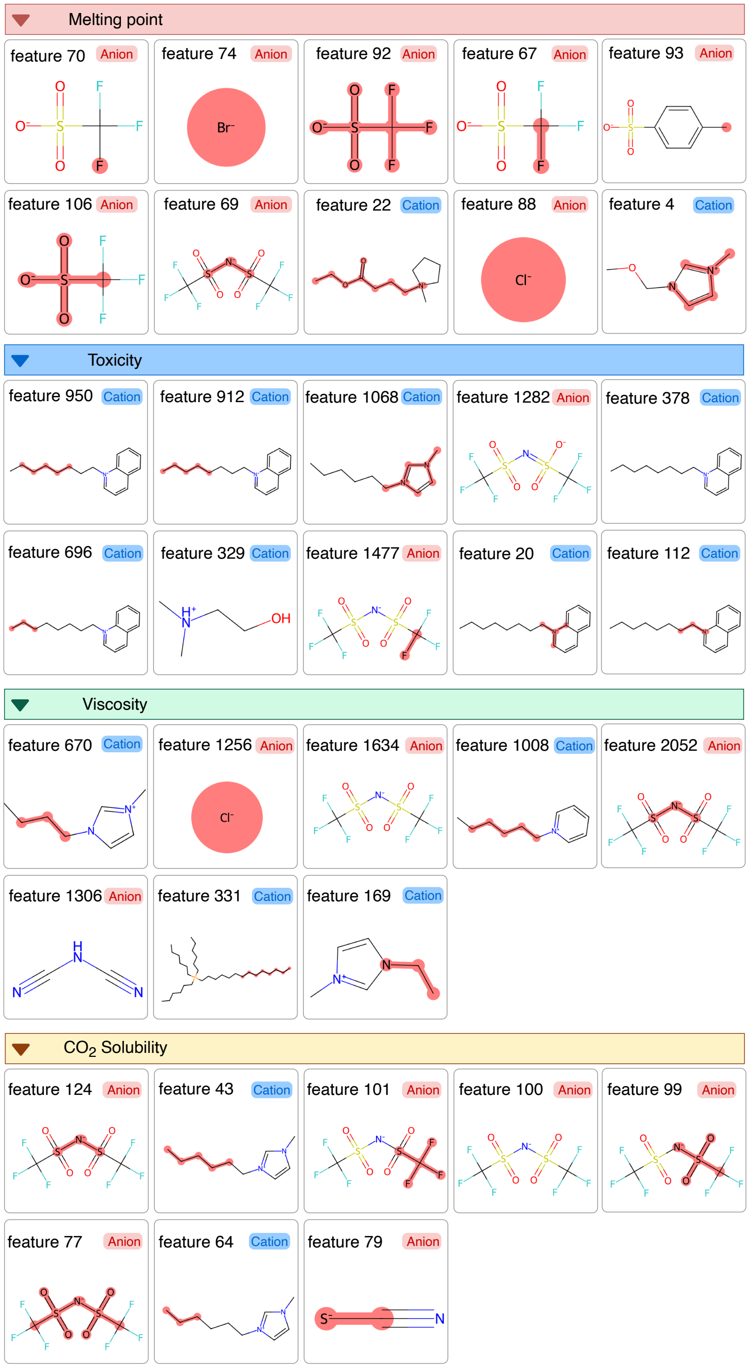

Figure S2: Top Morgan-fingerprint features by mean absolute SHAP value for each target-property model, displayed as 2D substructure depictions grouped by property: T_{m}, toxicity ($\log \mathrm{EC}_{50}$), viscosity ($\ln \eta$), and CO_2 solubility. Each tile is labeled with its fingerprint-bit index and tagged as cation (blue) or anion (red); red shading denotes a negative SHAP contribution and blue shading a positive one.

References

(1) Baldi, P.; Nasr, R. When is chemical similarity significant? The statistical distribution of chemical similarity scores and its extreme values. *Journal of chemical information and modeling* **2010**, *50*, 1205–1222.

(2) Bajusz, D.; Rácz, A.; Héberger, K. Why is Tanimoto index an appropriate choice for fingerprint-based similarity calculations? *Journal of cheminformatics* **2015**, *7*, 20.
